# Organic metal matrix Mil-88a nano-enzyme for joint repair in the osteoarthritis mouse model

**DOI:** 10.3389/fbioe.2023.1164942

**Published:** 2023-04-28

**Authors:** Hao Hu, Xu Huang, Yankun Dai, Kairun Zhu, Xuwen Ye, Shengdong Meng, Qing Zhang, Xueguan Xie

**Affiliations:** Huai’an Second People’s Hospital, Huaian, China

**Keywords:** nano-enzyme, osteoarthritis, mouse model, Mil-88a, organic metal

## Abstract

**Introduction:** In this paper we tried to conduct a novel nanomaterial strategy to overcome osteoarthritis (OA) in a mouse model.

**Methods:** In this regard, after synthesizing the Mil-88a nanozyme, as a certain Fe-MOF, its toxic effects were detected by CCK-8 method and live-dead staining. The OA model of mouse was constructed, and paraffin sections of joints were taken for histological evaluation. In addition, immunofluorescence and immunohistochemistry were used to identify the OA progression and OARSI was used to evaluate the OA grades. We observed that Mil-88a could be easily synthesized and has high biocompatibility.

**Results:** We observed that Mil-88a could significantly promote the expression of OA anabolism-related genes such as *Col2* and also significantly inhibit the expression of OA catabolism-related genes *MMP13*. Besides, we observed better OARSI score in animals treated with Mil-88a nano-enzyme loading on organic metal matrix.

**Discussion:** Overall, Mil-88a nano-enzyme could be used as a novel strategy to treat OA.

## Introduction

Osteoarthritis (OA) is also known as degenerative bone disease, and its clinical symptoms are usually manifested as joint swelling and pain, limited mobility, etc., and even lead to joint deformity and muscle atrophy (Wang et al., 2020). Clinically, the symptomatic treatment for OA is to mainly relieve pain and reduce deformity (Zhang et al., 2019). At present, a large number of basic research studies have carried out a closer understanding of the occurrence and development of OA, but its specific pathogenesis is still unclear. It is mainly believed that the most important reason for the pathogenesis of OA is the imbalance of reactive oxygen species (ROS) (Ding et al., 2020).

Reactive oxygen species (ROS) are known to be essential to cellular processes and are essential byproducts of cell metabolism, including superoxide anions (O_2_•), hydrogen peroxide (H_2_O_2_), and hydroxyl radicals (•OH) (Tsvetkov et al., 2022). ROS typically play a key role in the regulation of cell signaling pathways that normally control cell proliferation, growth, cycle, and death (Yi et al., 2021). ROS break the structure of signaling proteins by oxidizing redox-reactive residues on proteins, leading to the regulation of protein function (Gaurav et al., 2021). However, due to the oxidation of proteins, DNA, and membrane lipids, excess ROS may be a major cause of various inflammatory diseases (Zhai et al., 2021). Strategies to remove ROS by antioxidant molecules such as vitamins or natural enzymes such as superoxide dismutase (SOD) when the anti-ROS system is disrupted have a wide range of effects (Sun et al., 2018).

Spectroscopic capabilities have been shown to be effective biological therapeutic agents to combat oxidative stress in the treatment of various inflammatory diseases (Li et al., 2022). While there is promise in reducing ROS levels, many conventional antioxidant molecular treatments are less efficient than natural enzymes (He et al., 2020). At the same time, natural enzymes are subjected to many limitations, such as sensitivity to environmental conditions, limited functional stability, and difficulty in large-scale production (Li et al., 2017; Chen et al., 2018; Wan et al., 2020; Zhang et al., 2023). Recently, with the rapid and significant advances in nanotechnology, a series of biocatalytic or antioxidant nanostructures have been designed with unique ROS-scavenging capabilities, demonstrating promising activity to overcome these core challenges in clinical anti-ROS and anti-oxidation (Chen et al., 2020; Shetty et al., 2021). This is compared to conventional anti-inflammatory drugs, where biocatalytic or antioxidant nanostructures can achieve anti-inflammatory effects by eliminating a broad spectrum of reactive oxygen species, rather than targeting only specific inflammatory pathways or molecules, which may provide better therapeutic efficiency and greater biosafety than current clinical drugs (Zhang et al., 2018; Zhu et al., 2020; Cheng et al., 2021).

Thus, the idea of using biocatalytic or antioxidant nanostructures opens up new avenues for ROS clearance of ROS-related biotherapeutics, such as metal–organic framework (MOF) and polyphenol nanoparticles. Overall, biocatalytic or antioxidant nanostructures exhibit significant advantages, such as a remarkable ROS-scavenging capacity compared to natural enzymes, a broad spectrum of ROS elimination activity, robust stability in physiological environments, and satisfactory biocompatibility and biosafety (Li et al., 2020).

Eventually, we can regulate ROS levels to treat inflammatory diseases. It has also been reported that MOF nanoparticles have the ability to remove reactive oxygen species (Bian et al., 2020). ROS cleared by MOFs is a promising candidate for therapeutic applications due to its high biocompatibility, 3D matrix, and modification capabilities. Therefore, a novel, safe, and effective drug targeting ROS imbalance needs to be developed urgently. Nanozyme is a kind of nanomaterial with enzymatic activity similar to proteins. Mil-88a belongs to the family of metal–organic framework (MOF) nanozyme with peroxidase mimetic activity and good biocompatibility (Kasula et al., 2022) and thus is considered to be a promising nano-enzyme repair system for promoting OA repair. This study investigated the role of Mil-88a nanozyme co-loaded with the organic metal matrix in promoting OA repair in a mouse model.

## Materials and methods

### Material synthesis

The preparation process of Mil-88a was carried out based on the improved techniques of the existing literature (Gaurav et al., 2021; Yi et al., 2021). First of all, 0.9744 g (8.4 mmol) fumaric acid and 2.2722 g (8.4 mmol) FeCl_3_ were weighed. Then, the mixture was dissolved in 42 mL ultrapure water and stirred for 2 h. The solution was placed in the reaction kettle in a blast drying oven and then in a drying oven. The reaction was carried out at 85°C for 2 h. The reactor was taken out and cooled to room temperature. After the reaction kettle was cooled, the solution was centrifuged at 10,000 rpm for 10 min to obtain a brick-red solid. The precipitate was washed three times with absolute ethanol and dried in a vacuum drying oven at 100°C for 6 h. Ultimately, the solid obtained by drying was the metal–organic framework Mil-88a and was stored for future use.

### Characterization of Mil-88a

The aforementioned dried precipitate was used for SEM, XRD, TGA, and FTIR characterization. In addition, a small amount of the powder was ultrasonically dispersed in ultrapure water for particle size analysis by DLS.

### Detection of the SOD clearance rate of Mil-88a

Using the principle of the xanthine oxidase system to generate superoxide anion, according to the kit instructions (Beijing Solarbio Science & Technology Co., 100T/48S), the WST working solution and the enzyme working solution were prepared, respectively. A small amount of the sample powder was prepared as the mother solution, and then different proportions of the mother solution were mixed with pure water in a 1.5-mL tube to obtain sample solutions of different concentrations. The solution was incubated at 37°C for 20 min and was fully centrifuged to remove the material. The absorbance at 450 nm of the supernatant was measured, calculated, and compared with the control group, and the process was repeated three times for each group.

### Detection of the hydroxyl radical-scavenging rate of Mil-88a

Using the principle that hydroxyl radicals are generated by the Fenton reaction and the interaction between hydroxyl radicals and chromogenic reagents (BOXBIO, AKAO013M), different concentrations of Mil-88a solutions were prepared according to the kit instructions, and the hydroxyl radical-scavenging efficiency of Mil-88a at different concentrations was measured. The process was repeated three times.

### Primary culture of chondrocytes

Cartilage tissue was isolated from 5-day-old SD rats and washed with PBS containing 10% double antibody to remove blood clots. After incubation with 0.25% trypsin for 1.5 h at 37°C, 0.02% type II collagenase was replaced with 0.02% collagenase overnight to separate free cells. After the dissociated cells adhered to the wall, the medium was changed every 2 days and passed to the P3–P5 generation for use.

### Biocompatibility testing

CCK-8 method: A density of 5×10^4^ P3–P5 chondrocytes was inoculated in a 96-well plate overnight. After the cells adhered to the wall, the medium was changed and 100 μL, 1 μg/mL, and 2 μg/mL chondrocytes were added, respectively. Mil-88a was resuspended in 0.25 mL of fresh media to get different concentrations (0 μg/mL, 1 μg/mL, 2 μg/mL, 5 μg/mL, 10 μg/mL, 20 μg/mL, 50 μg/mL, and 100 μg/mL). After allowing culturing in the cell incubator for 24 h, the medium was completely removed and rinsed with PBS twice to completely remove the material. A measure of 110 μL of CCK-8 containing the medium prepared at a ratio of 1:10 was added and incubated at 37°C for 1 h. After incubation, the supernatant medium was carefully pipetted to a new 96-well plate, and the absorbance at 450 nm was measured using a microplate reader. Then, the process was repeated three times per set. Staining of live and dead cells: A total of 5,000 P3–P5 chondrocytes were seeded in a 96-well plate overnight, and then the original medium was replaced by 0.2 mL of the fresh medium containing different concentrations of Mil-88a (0 μg/mL, 1 μg/mL, 2 μg/mL, 5 μg/mL, 10 μg/mL, 20 μg/mL, 50 μg/mL, and 100 μg/mL) for 24 h. The medium was removed and washed two times with PBS. A measure of 100 μL of calcein-AM/PI staining working solution was added in the dark for 20 min, PBS was used to remove excess dye, and three fields of view were randomly selected for observation under a confocal microscope at ×100 magnification.

### PCR detection

A density of 3 × 10^5^ cells per well was inoculated in a six-well plate, and after culturing for 24 h, the old medium was removed and washed thoroughly with PBS, and then 1 mL of H_2_O_2_ induction solution prepared in a serum-free medium with a concentration of 450 μmol/L was added. After the cells were fully induced, equal volumes of Mil-88a solutions prepared with the serum-free medium of different concentrations were added, and culturing was continued for 24 h. Subsequently, the medium containing nanomaterials was removed by thorough rinsing with PBS. Ultimately, RNA was extracted for PCR experiments.

### Immunofluorescence

Each well was seeded with 75,000 chondrocytes of the same generation in a 24-well plate that had been placed on the slides. After adhering overnight, the cells were induced by oxidative stress in 0.25 mL of the serum-free medium mixed with H_2_O_2_ for 20 min. Then, the serum-free medium was replaced by 0.25 mL of the fresh medium containing different concentrations of Mil-88a (0 μg/mL, 1 μg/mL, 2 μg/mL, 5 μg/mL, 10 μg/mL, 20 μg/mL, 50 μg/mL, and 100 μg/mL). The treated cells were incubated for another 24 h, and it was washed three times with PBS buffer, fixed with 4% paraformaldehyde for 25 min, washed with PBS three times, then incubated with 3% H_2_O_2_ for 15 min, washed with PBS three times, and then incubated with goat serum for 30 min. After the blocking treatment, the diluted IL-1β primary antibody (1:200) was added dropwise to the slides and incubated overnight in a refrigerator at 4°C. Subsequently, the cells were incubated with biotinylated goat anti-rabbit secondary antibody (1:500) and allowed dilution for 1 h. Finally, the nuclei were labeled with 4′,6-diamidino-2-phenylindole (DAPI), incubated in the dark for 10 min, washed with buffer three times, dried the water on the slide, and sealed with neutral resin. After slicing, the sections were observed and photographed using a fluorescence microscope.

### OARSI score

In this study, the Osteoarthritis Research Society International (OARSI) score was used to evaluate the degree of articular cartilage degeneration (Chen et al., 2018). OARSI grade (grade) is divided into six grades, and the higher the score, the more serious the cartilage degeneration. Grade 0: normal, with normal cartilage surface and chondrocytes; grade 1: slight fibrosis on the articular cartilage surface, unevenness, but no cartilage loss, and no involvement but middle and deep cartilage; grade 2: fissures on the articular cartilage surface and cartilage loss, the middle layer of cartilage is involved, fibrosis, chondrocyte proliferation and death, abnormal cartilage matrix staining, etc.; grade 3: the fissure involves the deep cartilage, and chondrocyte death and proliferation are mainly distributed along the fissure; grade 4: the matrix around the fissure is lost and articular cartilage erosion; grade 5: articular cartilage erosion, full-thickness erosion of unmineralized hyaline cartilage, mineralized cartilage, and subchondral bone exposed on the articular surface; grade 6: joint deformation, articular surface fibrocartilage, osteophyte formation, etc. In this study, the OARSI score was independently completed by two sports medicine doctors with clinical work experience under the light microscope (×100) according to the scoring rules, and the average score was taken as the final score, and the sample grouping was not informed.

### Statistical analysis

SPSS and GraphPad Prism statistical software were used to analyze data, measurement data were expressed as mean ± standard deviation (xˉ ± s), and a *t*-test was used for the comparison between groups. Comparisons among multiple groups were performed using one-way analysis of variance (ANOVA). *p* < 0.05 was regarded as a statistically significant difference.

## Results

### Evaluating Mil-88a by scanning electron microscopy and characterization by XRD, TGA, and FTIR

**FIGURE 1:**
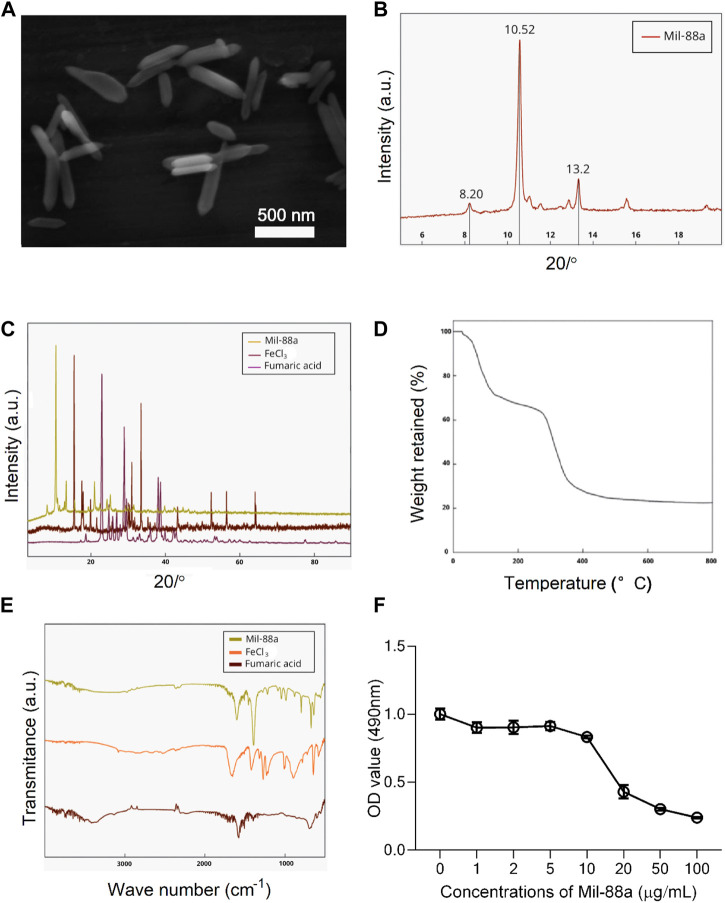
Characterization of Mil-88a. **(A)** Electron microscopy image of Mil-88a. **(B)** XRD characterization of Mil-88a. **(C)** XRD characterization of Mil-88a, FeCl_3_, and fumaric acid. **(D)** TGA characterization of Mil-88a. **(E)** FTIR characterization of Mil-88a. **(F)** Effects of different concentrations of Mil-88a on chondrocyte proliferation for 24 h.

Electron microscopy showed that the synthesized Mil-88a was in the shape of hexagonal rods, as shown in Figure 1A. The overall main peak of Mil-88a did not show a large shift in the whole spectrum. There were three characteristic peaks in the range of 6°–15°, which were 2.8°, 10.4°, and 12.9°, respectively, as shown in Figures 1B and C. The mass retention of Mil-88a was basically unchanged in the range of 100°C–300°C. FTIR showed that Mil-88a had all the characteristic peaks of FeCl_3_ and fumaric acid, as shown in Figures 1D, E. Next, the size of Mil-88a was characterized by dynamic light scattering (DLS) analysis, which demonstrated an ideal and uniform morphology with an average diameter of approximately 100 nm (Supplementary Figure S1).

### Biocompatibility evaluation of Mil-88a

**FIGURE 2:**
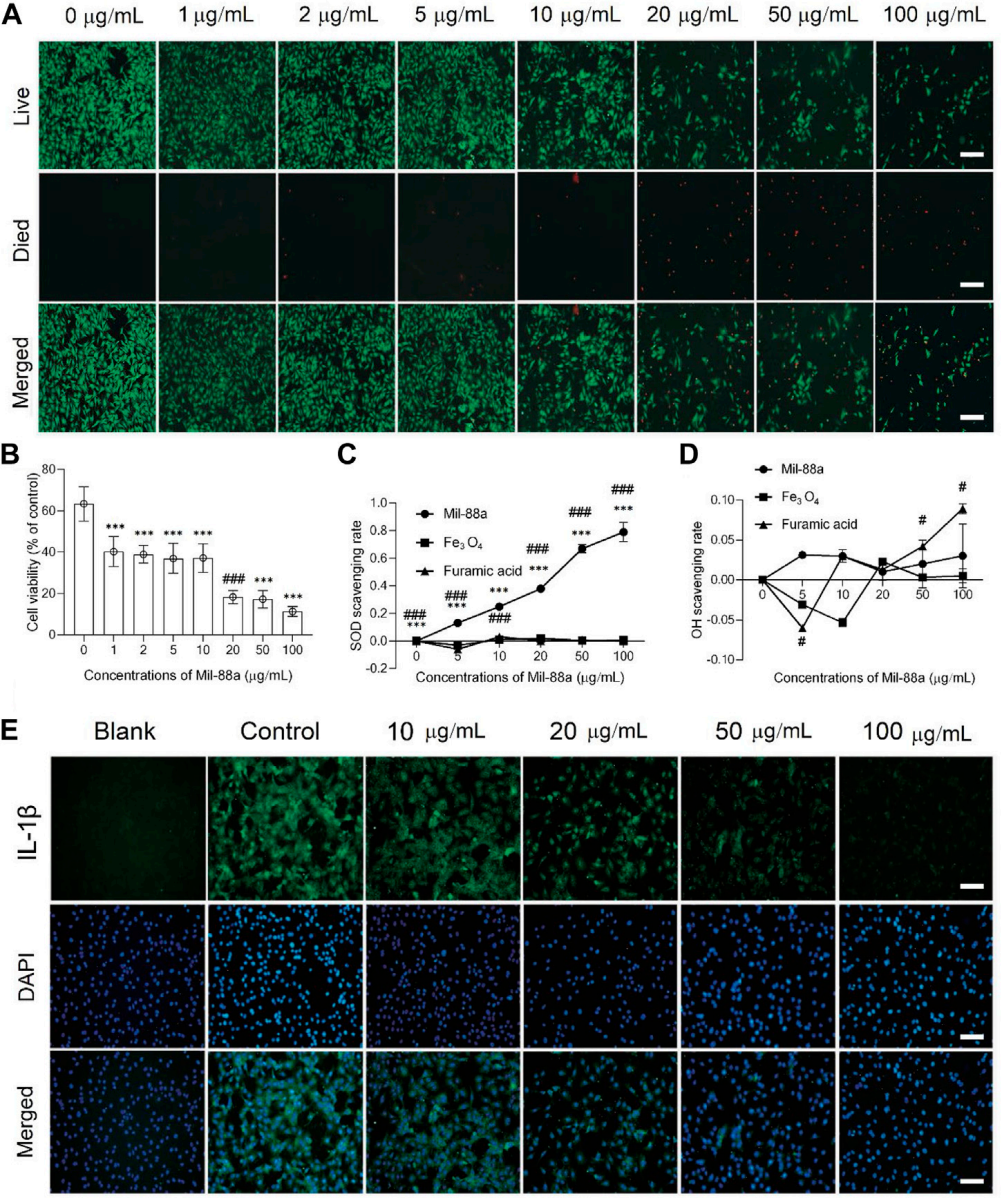
Cytotoxicity and biocompatibility testing of Mil-88a *in vitro*. **(A)** Fluorescence microscopy images of calcein-AM/PI staining in cells treated with different concentrations of Mil-88a (1, 2, 5, 10, 20, 50, and 100 μg/mL). Live, live cells (green); died, dead cells (red); merge, overlay. **(B)** Cell viabilities of chondrocyte cells treated with different concentrations of Mil-88a (1, 2, 5, 10, 20, 50, and 100 μg/mL and *n* = 4). The effects of different concentrations of Mil-88a (1, 2, 5, 10, 20, 50, and 100 μg/mL and *n* = 4) on SOD- **(C)** and OH- **(D)** scavenging rates for 24 h, and IL-1β **(E)**. ***p < 0.001 compared with 0 μg/mL; ###*p* < 0.001 compared with 10 μg/mL. Scale bar = 100 μm.

CCK-8 method: When the concentration of Mil-88a was in the range of 1–10 μg/mL, the chondrocyte survival rate was maintained above 80%, as shown in Figure 1F. Staining of live and dead cells: When the concentration was greater than 10 μg/mL, it showed strong toxicity to chondrocytes (*p* < 0.001), as shown in Figures 2A, B. ROS-scavenging effect of Mil-88a: With the increase in the Mil-88a concentration, the SOD-scavenging rate of Mil-88a gradually increased (*p* < 0.001; Figure 2C), and the hydroxyl radical-scavenging rate was different from that of Fe_3_O_4_, but the scavenging efficiency did not change with Mil-88a (*p* < 0.05, Figure 2D). This phenomenon takes place because when the nanoreactor reaches the tumor site, a high concentration of glutathione reduces Fe^3+^, triggering the structural collapse of MOF and the release of Fe^2+^, while GOx catalyzes the oxidation of glucose to H_2_O_2_. Then, the Fenton reaction occurs between H_2_O_2_ and Fe^2+^, producing the hydroxyl radical (•OH). Therefore, after Mil-88a, as a Fe-MOF, is taken up by cells, SOD is only consumed and •OH is consumed and increased. In addition, we evaluated the expression of IL-1β to figure out the effect of Mil-88a on pro-inflammatory cytokines (Figure 2E; Supplementary Figure S2). We observed that Mil-88a could significantly decrease the expression of IL-1β in a dose-dependent manner.

### Mil-88a alleviated the pathological manifestations of OA *in vivo*

**FIGURE 3:**
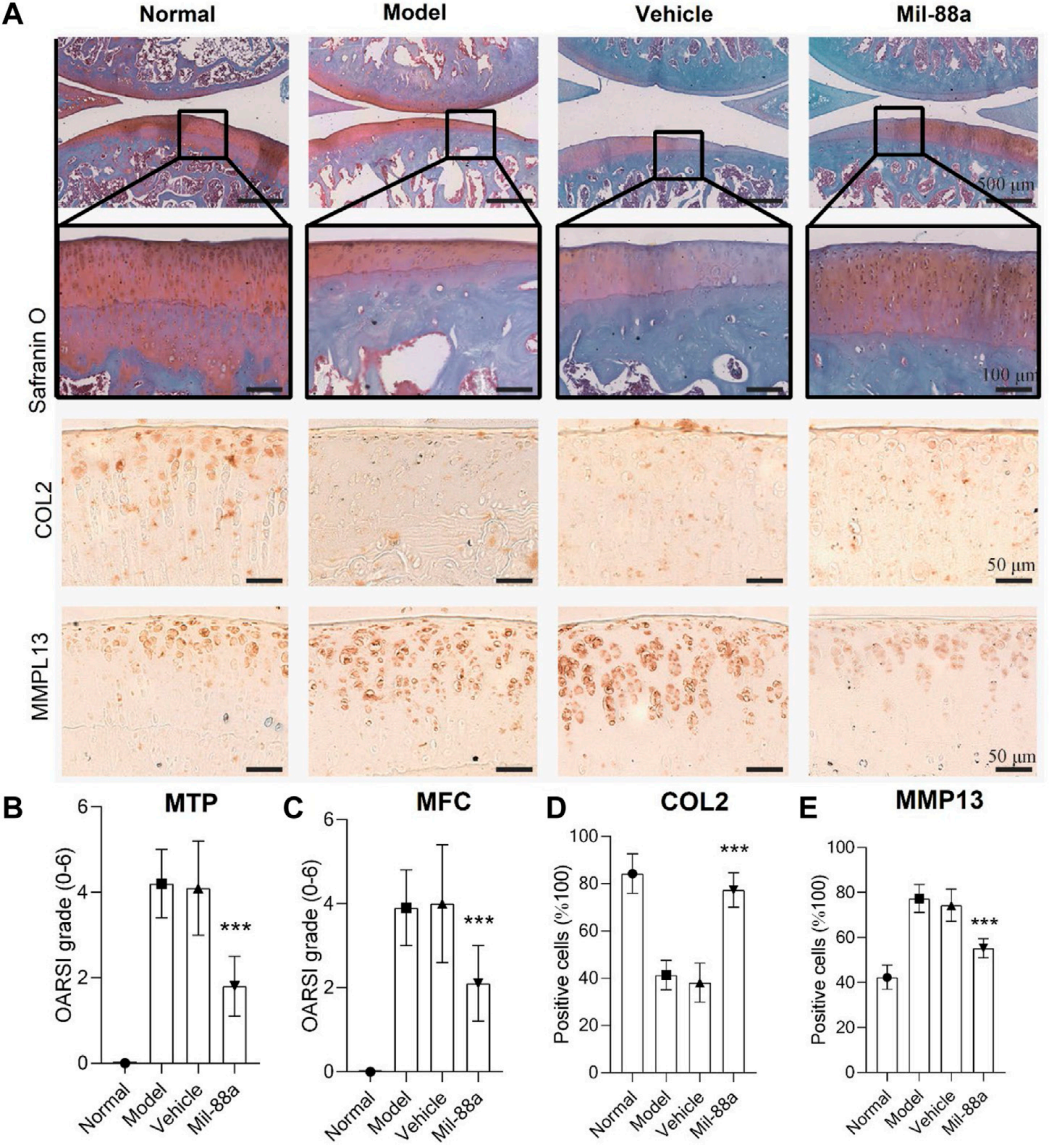
Mil-88a alleviates the pathological manifestations of OA. **(A)** Safranin O staining of paraffin sections of rat knee tissue, scale bar = 500 or 100 μm, and immunohistochemical staining of Col2 and MMP13, scale bar = 50 μm. **(B, C)** OARSI score. **(D, E)** Positive cell statistics of Col2 and MMP13. (*n* = 6). ns, no statistical difference; ∗*p* < 0. 05; ∗∗*p* < 0. 01; ∗∗∗*p* < 0. 001.

To explore the effect of Mil-88a nano-enzyme on the osteoarthritis mouse model, Mil-88a nano-enzyme was administered by local injection into the joint cavity. Safranin O staining of paraffin sections of rat knee joints showed that rats on a high-cholesterol diet showed a more severe OA phenotype than those on a normal diet (Figure 3A). The joint surface was not smooth, the safranin O staining and immunohistochemical staining intensity of type II collagen (col2) decreased, and the immunohistochemical staining intensity of MMP13 (matrix metalloproteinases 13) increased, indicating that the ECM proteoglycan content of articular cartilage Mil-88a nano-enzyme treatment can significantly increase the content of protein polysaccharide and type II collagen, effectively improve the degenerative lesions of the aforementioned knee cartilage tissue, and alleviate the progression of OA (Figure 3A). The severity of OA in the mouse was scored using the OARSI score. The results showed that the OARSI scores of MTP (Figure 3B) and MFC (Figure 3C) of the animals treated with Mil-88a were significantly lower than those of the animals in the non-treated group. This indicated that the degenerative lesions of the cartilage tissue in the Mil-88a-treated group were milder. Scoring of Col2 (Figure 3D) and MMP13 (Figure 3E) immunohistochemistry also showed this trend. The results suggest that Mil-88a could alleviate the progression of OA.

### Evaluation of the effect

**FIGURE 4:**
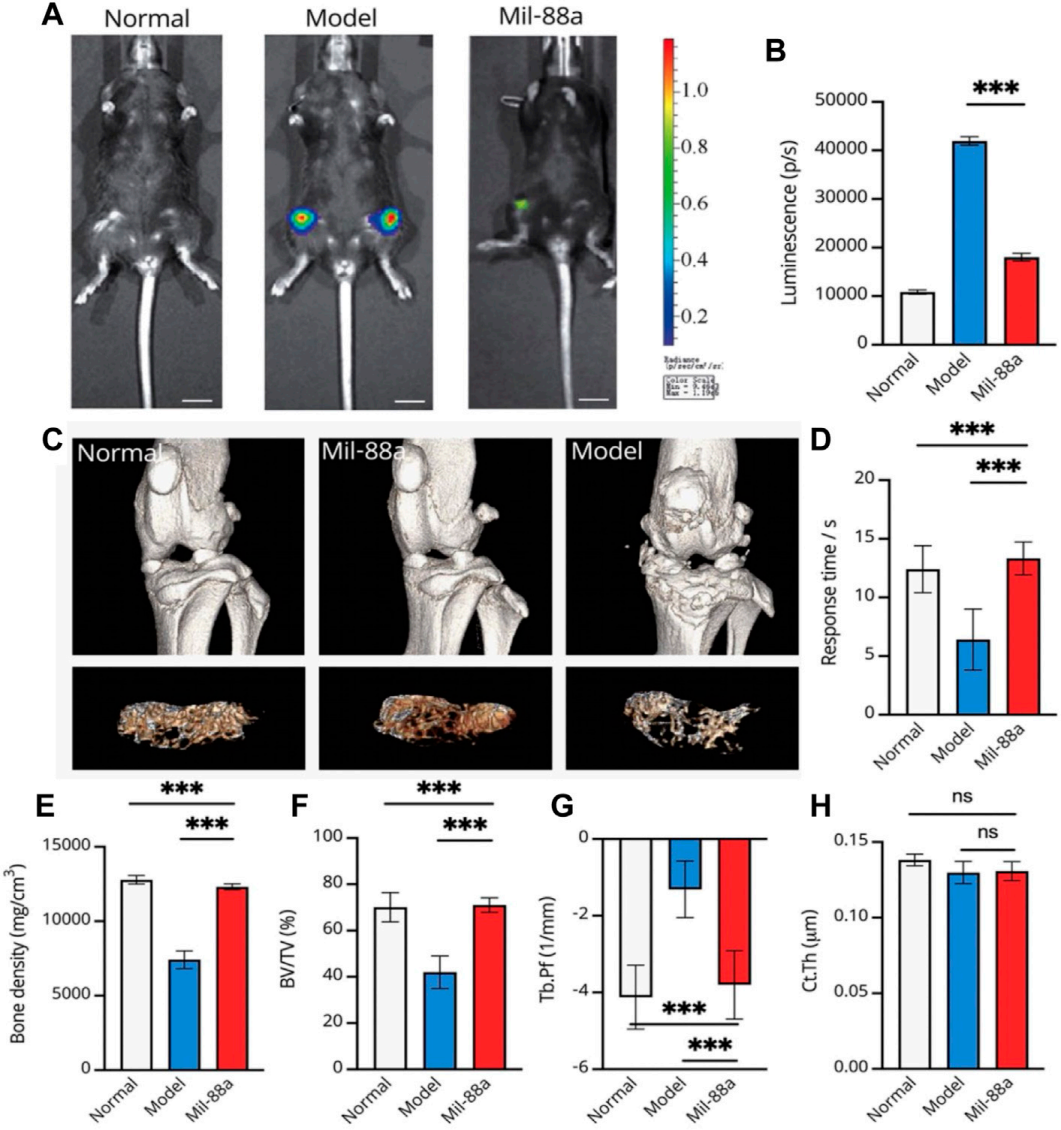
Representative luminescence imaging *in vivo* and evaluation of bone density in the mouse joint using micro-CT. Evaluation of osteoarthritis using luminescence imaging **(A, B)**. The 3D reconstruction of CT indicates the bone changes, and the tibial plateau section shows the structure of bone trabeculae **(C)**. For evaluating the response time, mice were placed on the heating platform at 55°C, and the reaction time of licking and jumping was shown; a micro-CT scan of the joints of mice was performed **(C, D)**. Bone density (mg/cm^3^) **(E)**, bone volume fraction (BV/TV) **(F)**, trabecular pattern factor (Tb.Pf, 1/mm) **(G)**, and cortical bone thickness **(H)** were used to assess subchondral bone changes in osteoarthritis by quantitative CT. Values were presented as mean ± SD. ∗∗∗*p* < 0.001 (*n* = 5).

**FIGURE 5:**
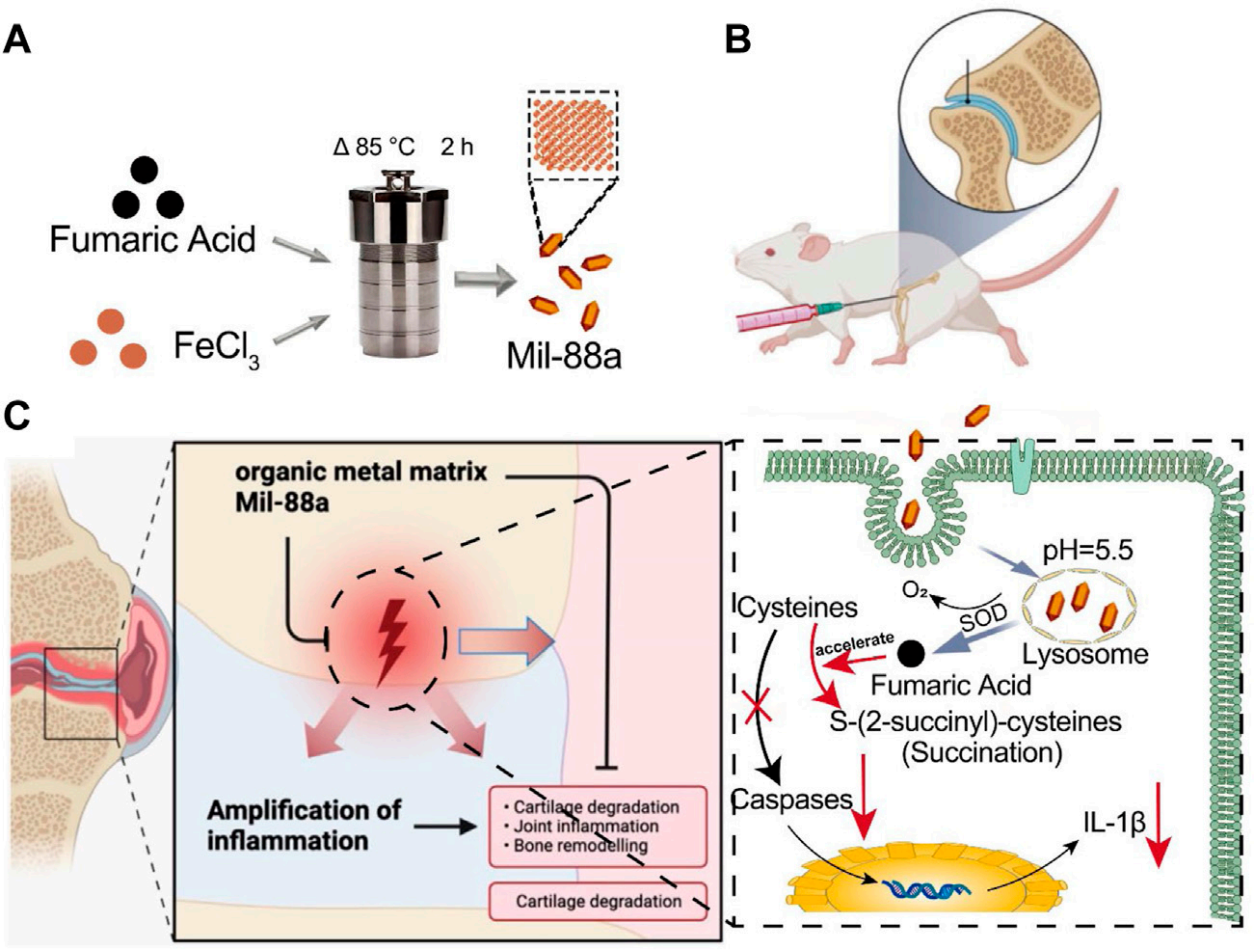
Schematic of how Mil-88a could improve OA in a mouse model. **(A)** Flow diagram of the prepared Mil-88a. **(B)** Illustration of the injected method of Mil-88a. **(C)** The treatment process for suppressing the expression of IL-1β and relief of inflammatory response.

In this section, we first tried to evaluate the conduction of OA in the mouse model using luminescence imaging (Figures 4A, B, 5). We observed high intensity of luminescence in the joints with the conduction of OA in comparison to normal mice (Figure 4B). In addition, we observed a significant drop in luminescence density in joints treated with Mil-88a. These results suggested that Mil-88a could improve OA *in vivo*. In addition, we applied a micro-CT scan to assess the effect of Mil-88a on bone density (Figures 4C, D). Dramatically, we observed that Mil-88a could increase bone density at the site of femoral joints (Figures 4E–H). These results suggested that Mil-88a could significantly increase bone formation to improve OA.

## Discussion

Approximately 16.2% of patients in China suffer from OA for a long time. The unbearable pain and extremely high disability rate seriously affect the quality of life of patients and destroy the stability of families and society (Xu et al., 2022). Although a lot of basic research has been carried out, the etiology and pathogenesis of OA are still inconclusive, and it is mainly believed to be related to factors such as age, occupation, strain, and endocrine (Lan et al., 2019; Qiu et al., 2020). Excessive ROS is the cause of arthritis inflammatory lesions. One of the important factors, as a potential target for the treatment of OA, is how to reverse the ROS imbalance, which has become the key to curing OA (Hou et al., 2021). MOFs are nanoparticles with a special 3D network structure formed by the self-assembly of metal nodes and organic ligands through chemical bonds. With a large specific surface area, adjustable pore size, and stable physicochemical properties, it is considered a promising biomimetic nanozyme (Xiong et al., 2022). Wu et al. (Zhou et al., 2022) reported that the special metal node structure of MOFs has oxidase analog activity, which is considered an ideal nanozyme for scavenging ROS. In this study, electron microscopy of Mil-88a showed a hexagonal rod shape, and XRD showed three characteristic peaks in the range of 6°–15°, which was consistent with the previous report (Chen et al., 2022). FTIR has all the characteristic peaks of raw materials, and TGA confirms that Mil-88a still has good stability in the range of approximately 100°C–300°C. In order to verify the cytotoxicity of Mil-88a, CCK-8 and live–dead staining indicated that when the concentration of Mil-88a was less than 10 μg/mL, there was basically no cytotoxicity (*p* < 0.001). In conclusion, Mil-88a was successfully synthesized with good biocompatibility. OA is mainly manifested as a cartilage defect. Reducing the oxidative stress of chondrocytes and removing excess reactive oxygen species will be beneficial to the regeneration of cartilage (He et al., 2021; Ma et al., 2022). ROS include SOD and OH (Chen et al., 2021). The ROS clearance rate of Mil-88a was further verified. The results showed that the SOD clearance rate of Mil-88 was concentration-dependent, and the OH clearance rate did not change with the concentration but was still higher than that of Fe_3_O_4_ (*p* < 0.05), suggesting that it is not simply due to its porous physical structure that scavenges ROS and the specific mechanism remains to be further studied. The results suggested that Mil-88a has the potential to act as a ROS-scavenging nanozyme in the joint cavity. ROS is an important signaling molecule that regulates inflammation. The imbalance of ROS leads to the abnormal expression of TNF-α and IL-1β inflammatory factors. Previous works of literature reported that TNF-α and IL-1β are closely related to cartilage destruction and the occurrence of synovitis (Ma et al., 2020; Cao et al., 2023). TNF-α, as a key inhibitor of cartilage collagen production, promotes the apoptosis of chondrocytes, and the activation of TNF-α causes the occurrence of the inflammatory cascade by upregulating the inflammatory trigger of IL-1β (Liu et al., 2021; Peng et al., 2021).

## Conclusion

In summary, we synthesized a novel platform based on Fe-MOF (Mil-88a) to overcome osteoarthritis (OA), which has good biosafety and can downregulate the expression of oxidative and inflammatory factors in OA-induced chondrocytes. In this regard, after synthesizing the Mil-88a nanozyme, its toxic effects were detected by the CCK-8 method and live–dead staining. The OA mouse model was constructed, and paraffin sections of the joints were obtained for histological evaluation. In addition, immunofluorescence and immunohistochemistry were used to identify the OA progression, and the OARSI score was used to evaluate the OA grades. We observed that Mil-88a could be easily synthesized and has high biocompatibility. We observed that Mil-88a could significantly promote the expression of OA anabolism-related genes such as *Col2* and also significantly inhibit the expression of OA catabolism-related genes such as *MMP13*. In addition, we observed better OARSI scores in animals treated with Mil-88a nano-enzyme loaded on the organic metal matrix. Overall, Mil-88a co-loaded with the organic metal matrix could be used as a novel nano-enzyme strategy in the treatment of OA. We also observed that Mil-88a nanozyme could significantly improve OARSI scores and OA in the mouse model in this study. Thus, our Mil-88a might be particularly meaningful and readily adapted to OA in a broad diversity of the current situation.

## Data Availability

The raw data supporting the conclusion of this article will be made available by the authors, without undue reservation.
