# Supplementary figures and images for: Organic metal matrix Mil-88a nano-enzyme for joint repair in the osteoarthritis mouse model
Source: Front Bioeng Biotechnol. 2023 Apr 28;11:1164942. doi: 10.3389/fbioe.2023.1164942 (PMC10175628; doi:10.3389/fbioe.2023.1164942)

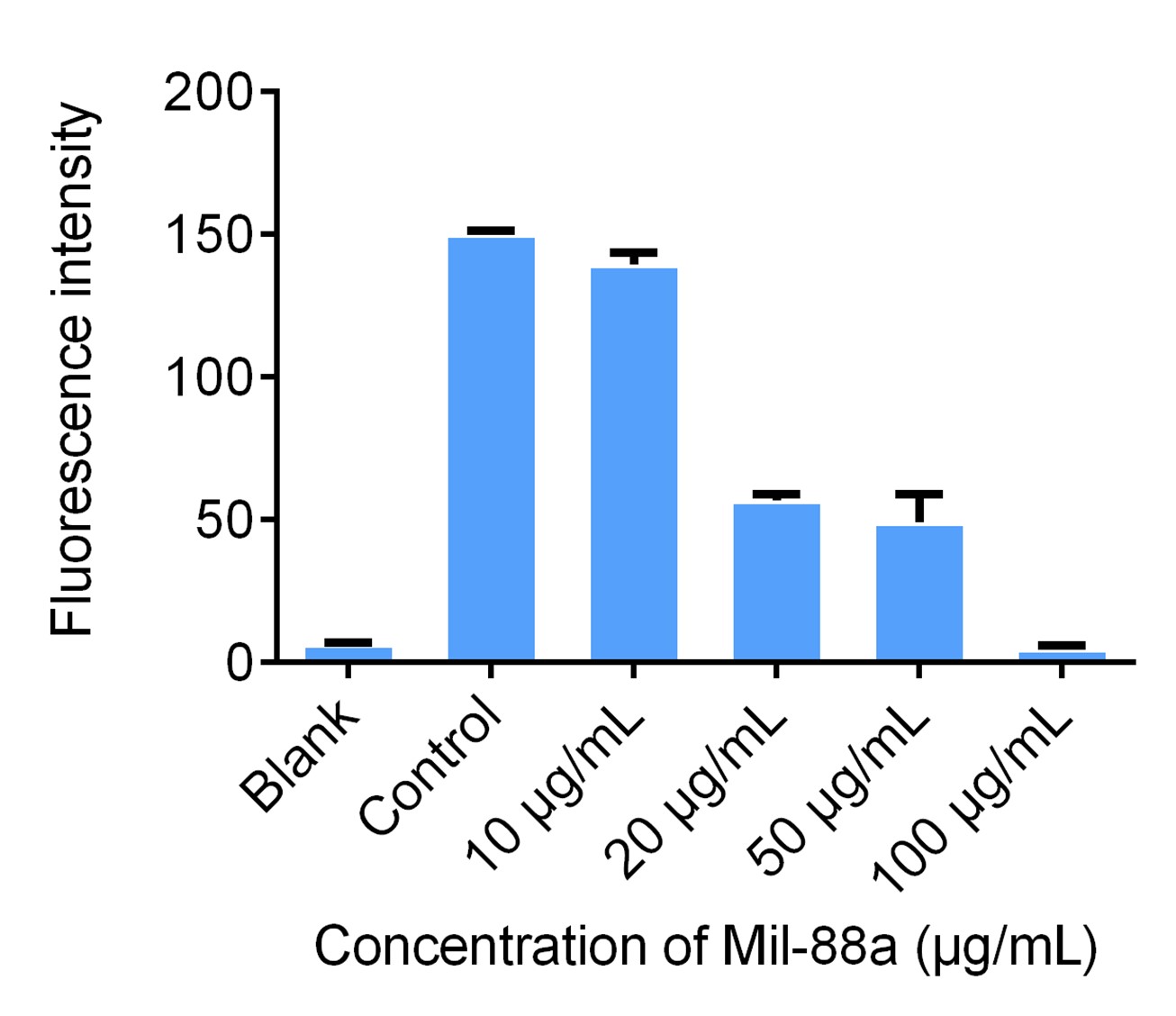

Supplement: Supplementary file 1 [file Image2.jpg]

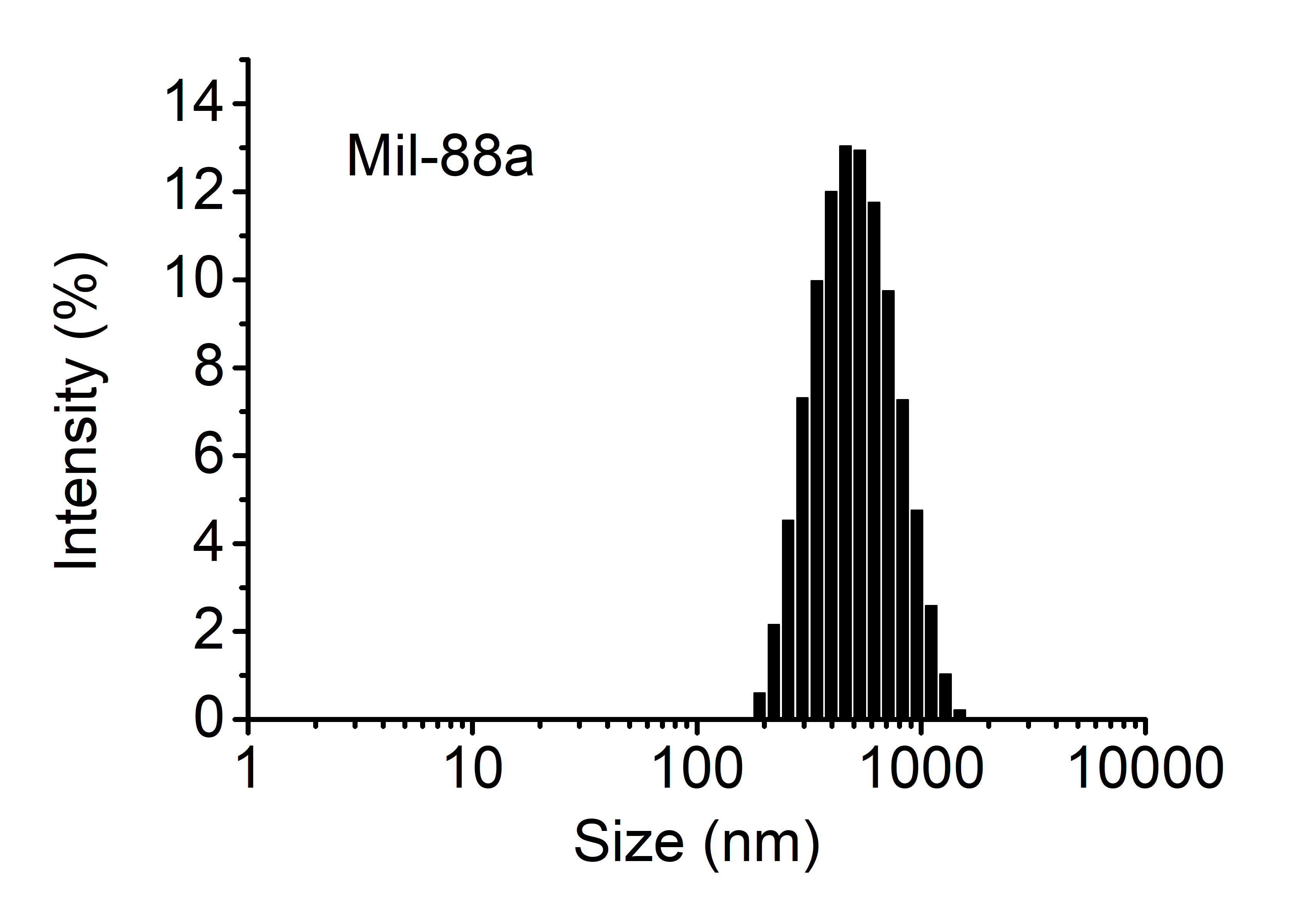

Supplement: Supplementary file 2 [file Image1.jpg]
